# Underwater Optics in Sub-Antarctic and Antarctic Coastal Ecosystems

**DOI:** 10.1371/journal.pone.0154887

**Published:** 2016-05-04

**Authors:** Pirjo Huovinen, Jaime Ramírez, Iván Gómez

**Affiliations:** 1 Instituto de Ciencias Marinas y Limnológicas, Facultad de Ciencias, Universidad Austral de Chile, Valdivia, Chile; 2 Centro Fondap de Investigación de Altas Latitudes (IDEAL), Valdivia, Chile; University of Shiga Prefecture, JAPAN

## Abstract

Understanding underwater optics in natural waters is essential in evaluating aquatic primary production and risk of UV exposure in aquatic habitats. Changing environmental conditions related with global climate change, which imply potential contrasting changes in underwater light climate further emphasize the need to gain insights into patterns related with underwater optics for more accurate future predictions. The present study evaluated penetration of solar radiation in six sub-Antarctic estuaries and fjords in Chilean North Patagonian region (39–44°S) and in an Antarctic bay (62°S). Based on vertical diffuse attenuation coefficients (K_d_), derived from measurements with a submersible multichannel radiometer, average summer UV penetration depth (z_1%_) in these water bodies ranged 2–11 m for UV-B (313 nm), 4–27 m for UV-A (395 nm), and 7–30 m for PAR (euphotic zone). UV attenuation was strongest in the shallow Quempillén estuary, while Fildes Bay (Antarctica) exhibited the highest transparency. Optically non-homogeneous water layers and seasonal variation in transparency (lower in winter) characterized Comau Fjord and Puyuhuapi Channel. In general, multivariate analysis based on K_d_ values of UV and PAR wavelengths discriminated strongly Quempillén estuary and Puyuhuapi Channel from other study sites. Spatial (horizontal) variation within the estuary of Valdivia river reflected stronger attenuation in zones receiving river impact, while within Fildes Bay a lower spatial variation in water transparency could in general be related to closeness of glaciers, likely due to increased turbidity through ice-driven processes. Higher transparency and deeper UV-B penetration in proportion to UV-A/visible wavelengths observed in Fildes Bay suggests a higher risk for Antarctic ecosystems reflected by e.g. altered UV-B damage vs. photorepair under UV-A/PAR. Considering that damage repair processes often slow down under cool temperatures, adverse UV impact could be further exacerbated by cold temperatures in this location, together with episodes of ozone depletion. Overall, the results emphasize the marked spatial (horizontal and vertical) and temporal heterogeneity of optical characteristics, and challenges that these imply for estimations of underwater optics.

## Introduction

The southeastern Pacific coast of the Chilean North Patagonia is characterized by a large and complex system of fjords and estuaries. This area coincides with the oceanographic transition between the sub-Antarctic and the cold-temperate zones, influenced by the Cape Horn Current and the Humboldt Current System, respectively. Regarding the biogeography, it is in the northern limit of the Magellan Province, with unique but still widely unexplored marine biodiversity [1–2]. Increasing knowledge on the oceanography (biological and physical) in this area has been gained in recent years (e.g. summaries of special issues [3–4]), however, gaps still remain in bio-optical characterization of these systems, which limits our knowledge on the factors related to, e.g. primary productivity and exposure to UV radiation of pelagic and benthic assemblages.

Previous studies in southern Chile and Antarctica have brought evidence on the potential of current levels of UV radiation in producing adverse effects on macroalgae [5–10]. However, despite of few reports including some information on UV penetration [5, 7, 9–12], to our knowledge, studies focused on bio-optical aspects in these aquatic environments are scarce [9, 13]. Underwater optics has received more attention in other marine [14] and in freshwater systems [15–16]. The role of dissolved organic matter (CDOM) in governing the attenuation of UV radiation, particularly in freshwaters, where the impact of catchment area is relatively stronger than in oceans, has been widely described [17]. In highly humic small lakes with high CDOM, penetration of UV-B wavelengths may be only few centimeters [18–20], while in low CDOM oceanic waters or clear oligotrophic lakes, where phytoplankton contributes more to the light attenuation, it can reach even dozens of meters [14, 21–22]. Knowledge on spectral differences in the attenuation is important for accurate evaluations of UV impact on organisms [23]. Spatial and temporal heterogeneity of bio-optical properties has been recognized as one of the challenges in larger-scale estimations [24–25].

Near-future scenarios for this sub-Antarctic region (belonging to the southern Austral Fjord Region, surface temperature 5–12°C) predict, e.g., seasonally enhanced freshwater runoff from melting of glaciers and increased rainfall [26]. Also direct human activities, such as intensive aquaculture industry, are leading to increased nutrient loading in these systems [27]. These anthropogenic perturbations could be expected to have a more severe local impact on areas with lower water exchange, such as fjords and inlets. Due to the geographic closeness, a risk related to the Antarctic ozone hole, extending also occasionally to southern parts of the South America, and the resulting increase in solar UV-B radiation with potential adverse impact on aquatic ecosystems is also of a concern in this region [28]. Thus, understanding underwater optics in natural waters is essential, not only in evaluating aquatic primary production and UV risk in aquatic habitats, but also for more accurate future predictions under current and future scenarios related with global climate change in these regions. Knowledge on light attenuation, and the potential impact of glacier-derived freshwater input in it, is also needed in explaining the observed spatial variations in primary production and carbon fluxes along Chilean Patagonia [29].

In the present study, underwater light penetration (UV and PAR) was examined in five zones in the North Patagonian fjord and estuary system (41–44°S) in southern Chile, including the estuaries of Yaldad and Quempillén rivers in the Island of Chiloé, the lower part of the Reloncaví Fjord, the Comau Fjord, and the Puyuhuapi Channel. Furthermore, one coastal site, the estuary of the Valdivia river (39°S), and Fildes Bay in the King George Island (62°S), Western Antarctic Peninsula, were studied. Spatial (horizontal and vertical) and in selected sites also seasonal (summer-winter) variation was evaluated. These seven localities, most including several measuring sites, represent coastal areas with different geomorphologies and environmental conditions, and in general with stronger impact from the catchment area than in open ocean. Based on studying these sites with different characteristics, we addressed the following main questions: 1) How does the underwater light climate (wavelength-specific attenuation and spectral proportions) and the risk of UV exposure vary across different types of estuarine and fjord systems and coastal waters in southern Chile and Antarctic? 2) How much does the underwater light penetration vary spatially (horizontally) within a study area (in scale of hundreds of meters or few kilometers)? 3) How optically heterogeneous/homogeneous is the water column with depth? 4) Does the underwater light climate vary seasonally? These aspects have relevance in the context of methodological issues (representativeness of measuring sites, calculations of diffuse vertical attenuation coefficients, K_d_), as well as in providing baseline information on underwater optics for different types of water bodies, with important implications for global climate change scenarios.

## Materials and Methods

### Study sites

**Fig 1:**
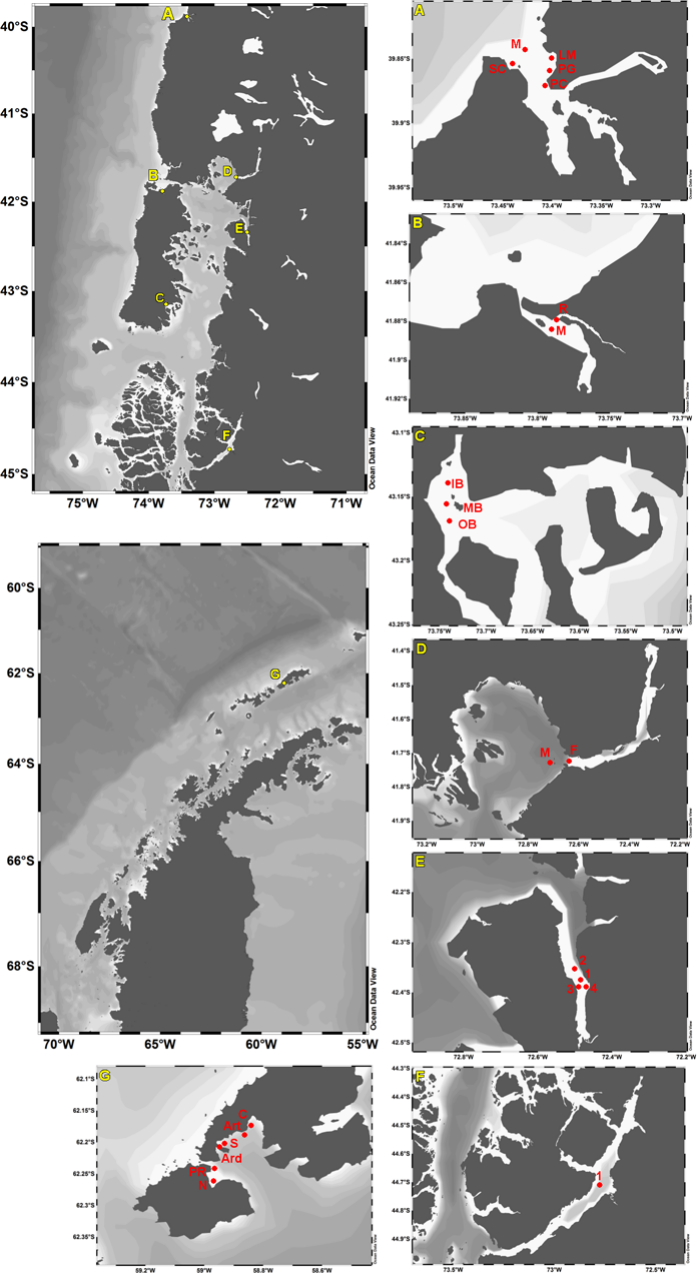
Map of study areas and sites of measurements in the Chilean North Patagonian region and Antarctica. Estuary of Valdivia river: Mouth (M), San Carlos (SC), Playa Chica (PC), Playa Grande (PG), Los Molinos (LM) (**A**). Estuary of Quempillén river: River (R), Mouth (M) (**B**). Yaldad: Inner bay (IB), Mid bay (MB), Outer bay (OB) (**C**). Reloncaví: Mouth (M), Fjord (F) (**D**). Comau Fjord (**E**). Puyuhuapi Channel (**F**). Fildes Bay: Nelson (N), Punta Rip (PR), Ardley (ARD), Shoa (S), Artigas (Art), Collins (C) (**G**).

Study areas were located along a latitudinal range between 39 and 62°S: the coast of Valdivia (estuary of Valdivia river) (39°S), Quempillén and Yaldad Estuaries in Chiloé Island, Reloncaví Fjord (41°S), Comau Fjord (42°S), Puyuhuapi Channel (44°S) and Fildes Bay in King George Island, Antarctica (62°S). Within most areas, several sites were included in order to estimate the spatial variation (**Fig 1**). The measurements were made in summer months and in some cases also in winter (**Table 1**).

**Table 1:** Study sites and dates of measurements. Information on ozone layer (based on OMPS; OMI for Valdivia March 2014; http://ozoneaq.gsfc.nasa.gov/tools/ozonemap/), solar zenith angle (SZA; calculated according to NOAA http://www.esrl.noaa.gov/gmd/grad/solcalc/; times of measurements given in S2 Data), total depth (based on echosounder, in deeper sites on bathymetric data by NOAA https://maps.ngdc.noaa.gov/viewers/bathymetry/), euphotic zone (z_1%PAR_ based on determined K_d_ values) and the number of measured profiles (n) are also given. NA = Not available.

Study site	Latitude / Longitude	Depth	z_1%PAR_	n	Date	Season	SZA	Ozone
		(m)	(m)				(°)	(DU)
**ESTUARIES**								
**Valdivia**								
Mouth	-39°50 S / -73°26 W	25	12	3	01.12.2014	Summer	21–33	285
San Carlos	-39°51 S / -73°26 W	16	>16	3	26.03.2014	Summer	42	277
Playa Chica	-39°52 S / -73°24 W	10	>10	3	26.03.2014	Summer	42–43	277
Playa Grande	-39°51 S / -73°23 W	10	>10	3	26.03.2014	Summer	44	277
Los Molinos	-39°50 S / -73°23 W	22	>22	4	26.03.2014	Summer	43	277
**Quempillén**								
River	-41°52 S / -73°45 W	2	>2	3	01.04.2015	Summer	46	294
Mouth	-41°52 S / -73°46 W	5	>5	3	01.04.2015	Summer	49	294
**Yaldad**								
Inner Bay	-43°08 S / -73°44 W	24	17	2	02.04.2015	Summer	60	286
Mid Bay	-43°08 S / -73°43 W	24	14	1	03.03.2014	Summer	52	291
Outer Bay	-43°09 S / -73°49 W	24	17	2	02.04.2015	Summer	62–63	286
**FJORDS**								
**Reloncaví**								
Mouth	-41°37 S / -72°57 W	215	17	2	20.03.2014	Summer	41	271
Fjord	-41°43 S / -72°34 W	474	17	2	21.03.2014	Summer	47–48	NA
**Comau**								
Site 1	-42°22 S / -72°25 W	>400	NA	1	19.12.2013	Summer (S1)	26	259
		>400	22	2	21.12.2013	Summer (S1)	37–40	265
		>400	8	1	28.08.2014	Winter	64	330
		>400	6	1	31.08.2014	Winter	56	318
Site 2	-42°21 S / -72°27 W	>400	17	2	02.12.2015	Summer (S2)	22	NA
Site 3	-42°23 S / -72°26 W	>400	19	2	02.12.2015	Summer (S2)	21	NA
Site 4	-42°22 S / -72°25 W	>400	18	2	02.12.2015	Summer (S2)	20	NA
**Puyuhuapi**								
Site 1	-44°41 S / -72°46 W	60	22	1	15.12.2014	Summer	22	293
		60	13	1	16.12.2014	Summer	22	289
		60	22	3	17.12.2014	Summer	24–30	277
		60	20	3	06.08.2015	Winter	61	325
**ANTARCTIC**								
**Fildes Bay**								
Nelson	-62°16 S / -58°58 W	150	34	2	01.02.2014	Summer	45–46	300
	-62°15 S / -58°57 W	150	19	1	13.02.2015	Summer	49	276
Punta RIP	-64°14 S / -58°58 W	35	26	2	13.02.2015	Summer	49	270
Ardley	-62°12 S / -58°57 W	21	>21	4	26.01.2014	Summer	43	275
Shoa	-62°12 S / -58°56 W	35	32	1	13.02.2015	Summer	49	276
Artigas	-62°11 S / -58°52 W	92	22	3	16.01.2014	Summer	41–46	300
		92	31	1	21.01.2014	Summer	42	308
Collins	-62°10 S / -58°51 W	58	29	2	21.01.2014	Summer	44–48	308

The study area in the coast of Valdivia (39°S, 73°W) has estuarine characteristics due to the influence of the Valdivia river. In this area, the variation in the regime of semi-diurnal tides is up to 2 m [9]. The estuary of the Quempillén river (41°52`S) is shallow with strong changes in its physico-chemical conditions (e.g. temperature, salinity) and turbidity through re-suspension of sediments and introduction of terrestrial and particulate material by tidal regimes, rain and wind, as well as exchange of fresh and sea water [5]. Estuary of Yaldad (43°08`S) is a shallow, marsh-like system where intensive aquaculture activities are present [30]. It has an area around 22 km^2^ and a maximum depth of 32 m, and it receives fresh waters from various rivers and streams. The regime of semi-diurnal tides ranges 3–5 m [31].

The large bay Reloncaví Sound (41°S, 72`W) has a maximum depth of 250 m and is subject to wide tidal fluctuations (up to 7 m). Strong winds and rain in winter and river runoff from e.g. the Reloncavi fjord, which receives freshwater from three rivers in addition to other diffuse sources, generates seasonal variation in the physic-chemistry of the water column [32]. Water quality is influenced by the turbidity due to sediment particles and seasonality in the wind conditions (both intensity and direction) [33]. Water characteristics are also affected by discharges of organic matter from intensive aquaculture activities and from the city of Puerto Montt [34].

The Comau Fjord in the northern part of the Chilean Patagonia (42°22`S) is over 30 km long and connected to the Gulf of Ancud via the Comau Channel [35]. It is deep (max. 500 m) and surrounded by mountains up to 2000 m altitude. Interaction of nutrient-rich sub-Antarctic waters and freshwater from seasonal snowfall, rain and rivers generates strong stratification of the water column [35–36]. Lack of glacier influence results in less fine inorganic sediments than in other southern fjords [37]. Also here the climate can be characterized by strong seasonality with dry summer and rainy winter [32].

Puyuhuapi Channel forms part of a complex channel system, and is connected to larger Moraleda (south) and Jacaf (north) Channels. Ventisquero Sound, the northern part of the channel, is divided by the Galvarino Pass, and receives freshwater from the Veintisquero River, while the Cisnes River discharges into the southern part. Puyuhuapi Channel exhibits strong seasonal variation with more haline surface waters towards the southern part in summer, while in winter the intrusion of oceanic waters through Jacaf Channel can result in reverse pattern [38]. High vertical stratification of water column in summer and partially mixed in winter is a characteristic more pronounced towards northern part. In Puyuhuapi Channel severe hypoxic conditions have been reported [38].

Fildes Bay (or Maxwell Bay) is a 19-km long inlet in King George Island (South Shetland Islands, Antarctica), surrounded by Collins Glacier (or Bellingshausen Glacier Dome), the Nelson Island and the Fildes Peninsula (**Fig 1**). The island is largely covered by permanent ice and glaciers, however, in summer period ice-free coastal areas exist. Overall extreme physical conditions as well as perturbations from ice-driven physical processes (e.g. icebergs) and sea ice formation (around 2–5 m) are characteristic. King George Island belongs to the maritime Antarctica eco-region, which presents relatively higher temperatures and precipitations than in the continental regions [39].

The studies in the Antarctic were carried out under permission granted by Instituto Antártico Chileno (INACH) in accordance with the Protocol on Environmental Protection to the Antarctic Treaty. The study did not involve Antarctic Specially Protected Areas (ASPAs), and sampling of protected or endangered species.

### Underwater light measurements

The measurements of underwater solar radiation were carried out with the multichannel radiometer PUV-2500 (Biospherical Instruments Inc., USA) connected to computer with Profiler software. The instrument measures wavelengths 305, 313, 320, 340, 380, 395 nm and PAR waveband (400–700 nm) as well as temperature. The angular response of the sensor is ±2% in 0–65°. The measurements of underwater light profiles were carried out within few hours around solar noon (between 11:00–16:00 h; **S2 Data**) in order to minimize the impact of solar zenith angle (**Table 1**), and during sunny or partly cloudy days, with calm or moderate wave conditions. Prior to starting the measurement of a profile, initial dark readings were measured by maintaining the optical sensor in the darkness for 45–60 s. After that the instrument was lowered in the water column at a steady rate down to maximum of 25 m (cable length). Only values obtained while lowering the instrument in the water column were used. Attention was paid to the orientation of the boat during the profile in order not to create shadow on the instrument.

In optically homogenous water column solar irradiance attenuates with depth according to equation E_d_(z) = E_d_(0)e^-Kdz^, K_d_ being the vertical diffuse attenuation coefficient for downward irradiance, E_d_(z) the downwelling irradiance at depth z, and E_d_(0) the irradiance right below the surface [40]. K_d_ values were calculated from the slope of the linear regression of the natural logarithm of irradiance vs. depth for each wavelength. They were processed manually using Excel platform (allowing removing noise and data anomalities), and were also contrasted with the data output of Profiler software in order to ensure the accuracy. The homogeneous upper water column down to a depth (strongly depending on the wavelength; for PAR on an average 3–22 m in different studied water bodies) where the curve remained log-linear (r^2^ ≥ 0.90) was the depth range used for the calculation. In cases where marked variation in irradiance values was observed near the surface and in the upmost water column, mainly due to surface waves, a depth where the variation was no longer observed was used as the upper limit of the depth profile (focal depth; [41]). This depth ranged on an average 0.5–3 m in different studied water bodies (max. 8 m), and was more pronounced during measurements in Antarctica, Puyuhuapi Channel and Reloncaví Fjord, and in general affecting mainly longer wavelengths (395 nm and PAR). K_d_ values for each wavelength were used to estimate a depth where 1% and 10% of the sub-surface irradiance reach (z_1%_ = 4.6/K_d_, z_10%_ = 2.3/K_d_; Kirk 1994). In the case of PAR z_1%_ is used as an estimation of euphotic zone. In order to analyze the wavelength-dependence of K_d_, the slope of ln K_d_ vs. wavelength (305–395 nm) curve (S_Kd (305-395nm)_) was plotted against wavelength (305–395 nm). In addition, K_d_ values for each one-meter water column were determined in a similar way as described above, but using one-meter depth ranges (e.g. 0–1 m, 1–2 m etc.). Spatial (vertical and horizontal) variation of these K_d_ values was visualized with Ocean Data View software (Schlitzer R., Ocean Data View, odv.awi.de, 2015).

### Statistical analyses

Prior to analysis, a test of homoscedasticity of variance was carried out. Due to that the data did not show homocedasticity according to Cochran’s test, the comparison of z_1%_ (313 and 395 nm) between the seven study areas was conducted applying the Kruskal-Wallis test. To classify differences between means of S_Kd305-395nm_, a Levene´s test was performed for one-way ANOVA followed by a post hoc Newman-Keuls test. Box-Cox transformation was used to meet normality.

For the comparison of the differences in K_d_ values between sites and seasons, multivariate Repeated Measures ANOVA was used. Two localities with the highest numbers of measuring sites (Valdivia and Fildes Bay) and one locality where three seasons were examined (Comau fjord) were selected. In this case K_d_ values for different wavelengths were considered within subject effects (seven levels), while sites or seasons were regarded as the main effects. Sphericity was tested using the Mauchly’s test and the multivariate test methods Willks and Pillai’s were applied. Post-hoc comparison of means for the interaction wavelength x sites and wavelengths x seasons were assessed using a Tuket LSD test. In the case of the Puyuhuapi Channel, which was studied in summer 2014 and winter 2015, the differences between seasons were tested using multivariate Hotteling’ s T^2^ test. All the analyses were carried out with the software Statistica 7 (StatSoft, Inc, USA).

In order to test which variable or suite of optical variables allow classifying the different study areas, a multivariate discriminant function analysis was performed. K_d305_, K_d320_, K_d340_, K_d380_, and K_dPAR_ as well S_Kd305-395nm_ were entered as the dependent variables in the multivariate matrix. The significance of the model and the individual contribution of each variable to the discrimination between groups were estimated using Wilks’ lambda and partial Wilks’ lambda (ranging from 0 to 1 where 0 denotes perfect discriminatory power). Thereafter, a canonical analysis based on discriminating coefficients (canonical functions), eigenvalues, cumulative proportions, and score representation of the discriminant functions was performed in order to classify the variables that contribute most to the discrimination between localities.

## Results

### North Patagonian estuaries

**Fig 2:**
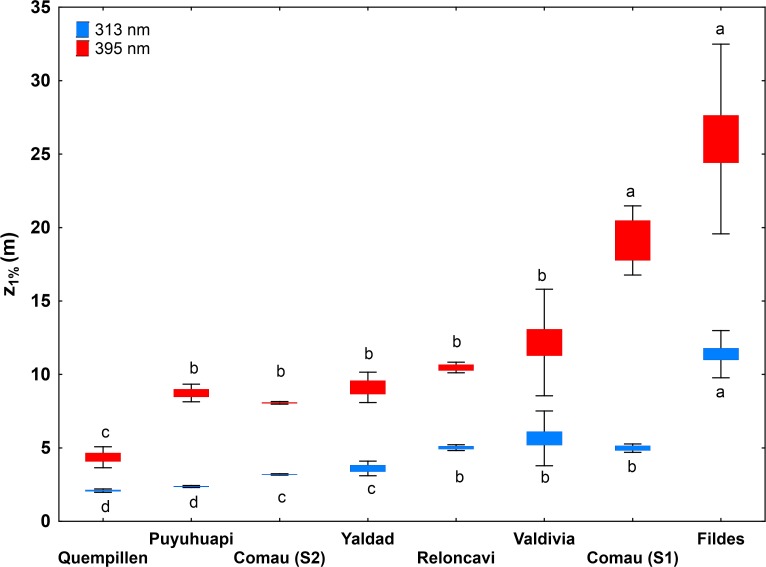
Comparison of the 1% summer penetration depth (z_1%_) of UV-B (313 nm) and UV-A (395 nm) radiation in the North Patagonian region and Antarctica, based on K_d_ derived from the optically homogeneous upper water column. For Comau Fjord two different summers (S1 = 2013, S2 = 2015) are presented separately (**S1 Data**). Values are means ± S.D., n = 16 (Valdivia), 6 (Quempillén), 5 (Yaldad), 4 (Reloncaví), 3 (Comau S1), 6 (Comau S2), 5 (Puyuhuapi), 16 (Fildes). Different letters indicate significant differences between the study sites (Results of Kruskal-Wallis test and Neuman-Keuls post-hoc are indicated in **S1A Table**).

The 1% summer penetration depths (z_1%_) of UV radiation, corresponding to the longest measured wavelengths UV-B (313 nm) and UV-A (395 nm) thus indicating the lower limits for UV penetration, in the studied locations are summarized in **Fig 2**. The estuary of Quempillén river showed the strongest UV attenuation of all the study locations (K_d313nm_ 2.1–2.3 m, K_d%395nm_ 0.9–1.2 m). The other two estuaries did not vary significantly in their UV-A penetration (z_1%395nm_ 6.9–15.9 m for Valdivia, 8.3–14.7 m for Yaldad), while UV-B penetrated deeper in the estuary of Valdivia river (z_1%313nm_ 3.6–8.1 m) than in Yaldad (z_1%313nm_ 3.4–4.7 m) (**Fig 2; S1A Table**).

**Fig 3:**
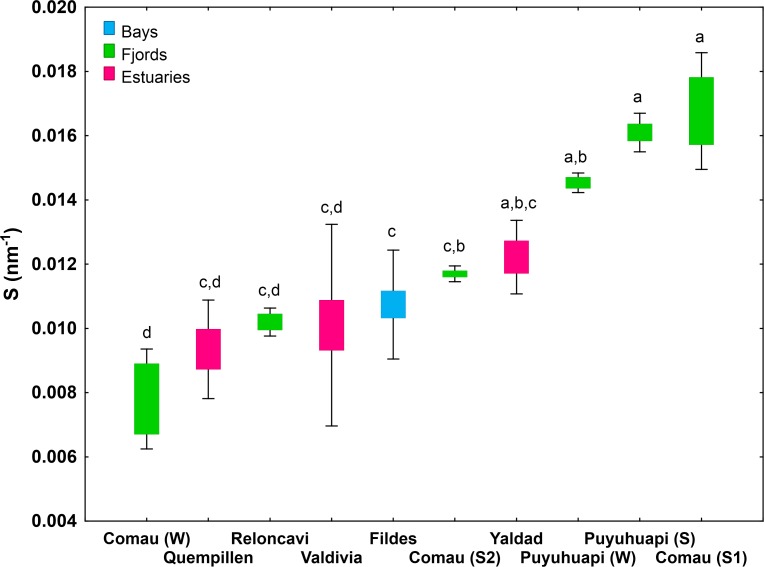
Slope of the curve ln K_d_ vs. wavelength (305–395 nm) in the North Patagonian region and Antarctica. For Comau Fjord two different summers (S1 = 2013, S2 = 2015) and winter 2014 (W), and for Puyuhuapi Channel Summer 2014 (S) and Winter 2015 (W) are presented separately (**S1 Data**). Values are means ± S.D., n = 16 (Valdivia), 6 (Quempillén), 5 (Yaldad), 4 (Reloncaví), 3 (Comau S1), 6 (Comau S2), 2 (Comau W), 5 (Puyuhuapi S), 3 (Puyuhuapi W), 16 (Fildes). Different letters indicate significant differences between the areas (Box-Cox transformation, ANOVA and Newman-Keuls post hoc are indicated in **S1B Table**).

**Fig 4:**
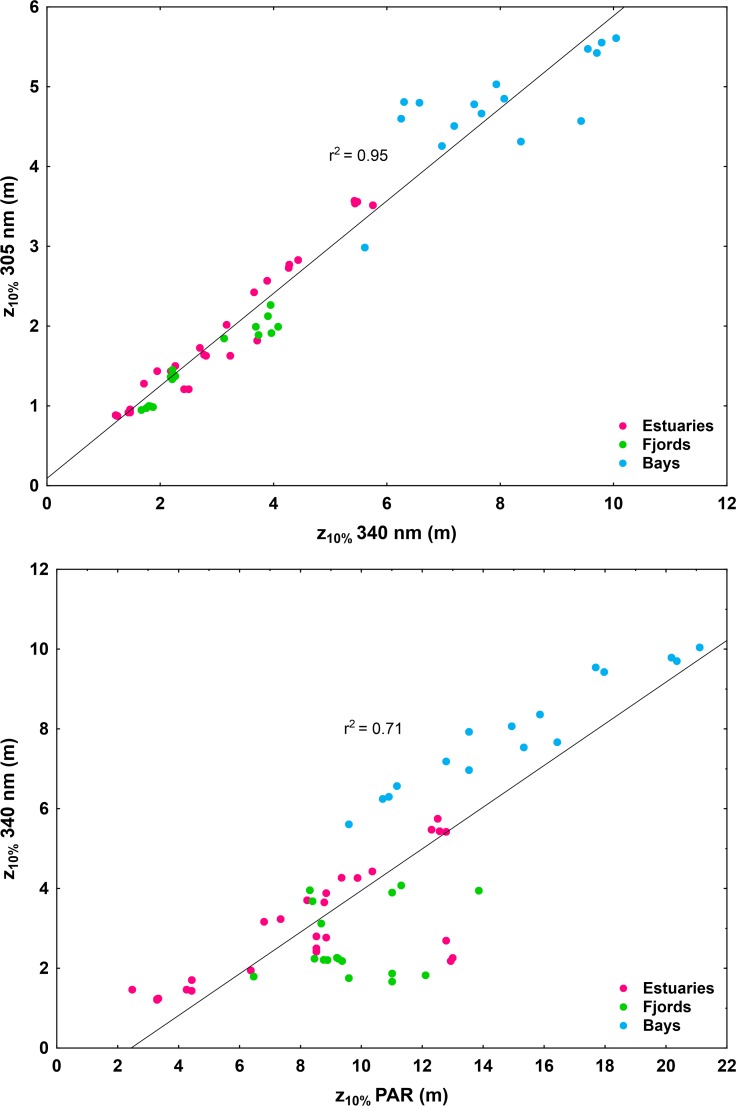
The relationship between the 10% penetration (z_10%_) of UV-B (305 nm) and UV-A (340 nm) as well as of UV-A (340 nm) and PAR (400–700 nm) in the North Patagonian estuaries and fjords and Antarctic bay. Individual replicate values are presented for all the studied dates (**S1 Data**).

The wavelength-dependence of K_d_ (S_Kd (305-395nm)_ ranging 0.0078–0.0111 nm^-1^ in Quempillén, 0.0071–0.0208 nm^-1^ in Valdivia, 0.0112–0.0133 nm^-1^ in Yaldad) did not vary significantly between the studied estuaries (p> 0.05; **Fig 3; S1B Table**). UV-A (340 nm) penetrated (z_10%_) 1.5–1.9 times deeper than UV-B (305 nm) in the studied estuaries, while the z_10%_ of PAR was 2.6–3.0 times higher than of UV-A (340 nm) (**Fig 4**).

**Fig 5:**
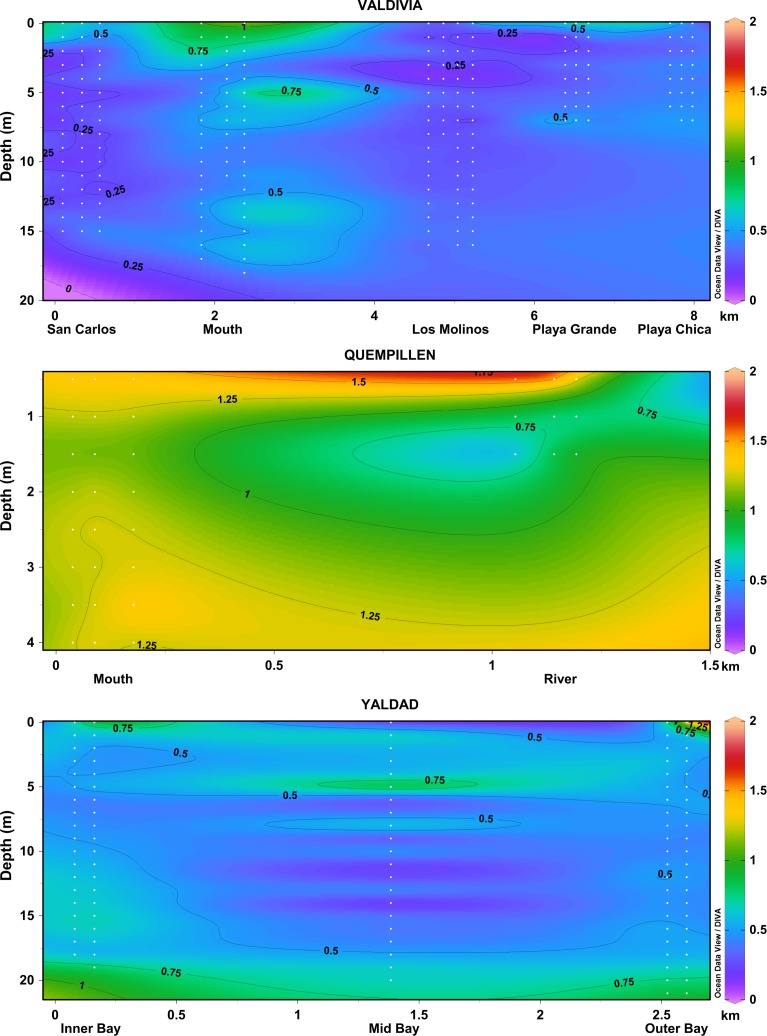
Vertical variation of K_d395nm_ (presented for each one or half-meter water layer) in the studied estuaries in the North Patagonian region, visualized with Ocean Data View software (Schlitzer R., Ocean Data View, odv.awi.de, 2015). The data of different sites of measurements are included in order of their geographical location (see map in **Fig 1**; distances not in scale), including all the individual measurements (white dots indicating the measured values; **S2 Data**).

**Fig 6:**
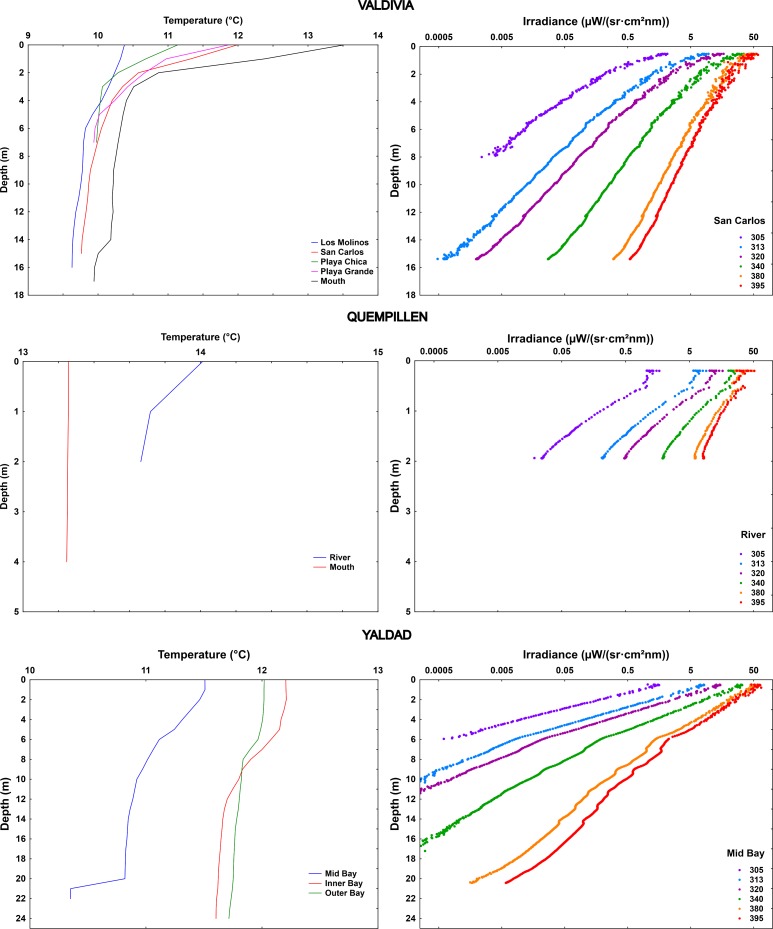
Vertical profiles of temperature (mean of replicate measurements for each location) and underwater solar UV radiation (305–395 nm) (representative example of one site) in the studied estuaries in the North Patagonian region.

The estuary of Valdivia river was characterized by spatial (horizontal and vertical) variation in the underwater attenuation of solar radiation (**Fig 5**). The multivariate Repeated Measures ANOVA revealed that K_d_ values in different sites were significantly different (p<0.001), and the interaction between sites and wavelengths was evident (**S2A Table**). The highest K_d_ (both for UV and PAR) was observed in the study site at the mouth of the river (p>0.05; Tukey LSD with strong thermal and optical stratification (**Figs 5 and 6**). San Carlos also showed high UV attenuation. The lowest K_d_ for UV was observed in Los Molinos and Playa Grande (p>0.05, Tukey LSD; **S2A Table**) where the water column was also optically more homogeneous along a depth gradient.

The estuary of Quempillén river showed the strong attenuation of solar radiation (**Fig 5**). In the shallow river site the observed optical stratification coincided with the thermal stratification (**Figs 5 and 6**). In the mouth of the river, the entire water column was mixed and optically more homogeneous (**Figs 5 and 6**).

In Yaldad, the sites in the middle of the bay (mid bay) and closest to the river (inner bay) showed optical stratification coinciding with the thermal stratification, with higher K_d395nm_ observed below the mixed layer or thermocline (**Figs 5 and 6**). Towards more open area (outer bay) less optical and thermal stratification was observed within the water column, with the exception of the immediate surface water layer (**Figs 5 and 6**).

### North Patagonian fjords

The studied fjords did not differ significantly in their summer UV-A penetration depths (z_1%395nm_ 8.7 m for Puyuhuapi, 8.1 m for Comau (summer 2015), 10.4 m for Reloncaví), with the exception of Comau Fjord in summer 2013 (z_1%395nm_ 19.1 m). UV-B attenuated more strongly in Puyuhuapi Channel (z_1%313nm_ 2.4 m) than in Reloncaví (z_1%313nm_ 5.0 m) and Comau (z_1%313nm_ 3.2 summer 2015; 5.0 m summer 2013) fjords (**Fig 2; S1A Table**). Puyuhuapi Channel (0.0142–0.0166 nm^-1^) together with Comau Fjord in summer 2013 (0.0153–0.0188 nm^-1^) showed the highest values of S_Kd (305-395nm)_ of all the studied locations. S_Kd (305-395nm)_ in Comau Fjord in summer 2015 (0.0113–0.0120 nm^-1^) and in Reloncaví Fjord (0.0099–0.0107 nm^-1^) did not differ significantly from the studied estuaries and Antarctic bay (**Fig 3; S1B Table**). Summer z_10%_ for UV-A (340 nm) was 1.7–1.8 times deeper than for UV-B (305 nm) in all the studied fjords, however, more variation in z_10%PAR_/z_10%340_ was observed: in Reloncaví Fjord z_10%PAR_/z_10%340_ (2.4) was in the range of the studied estuaries, while in Comau Fjord (3.7) and Puyuhuapi Channel (5.6) UV-A attenuated more rapidly in proportion to PAR (**Fig 4**).

**Fig 7:**
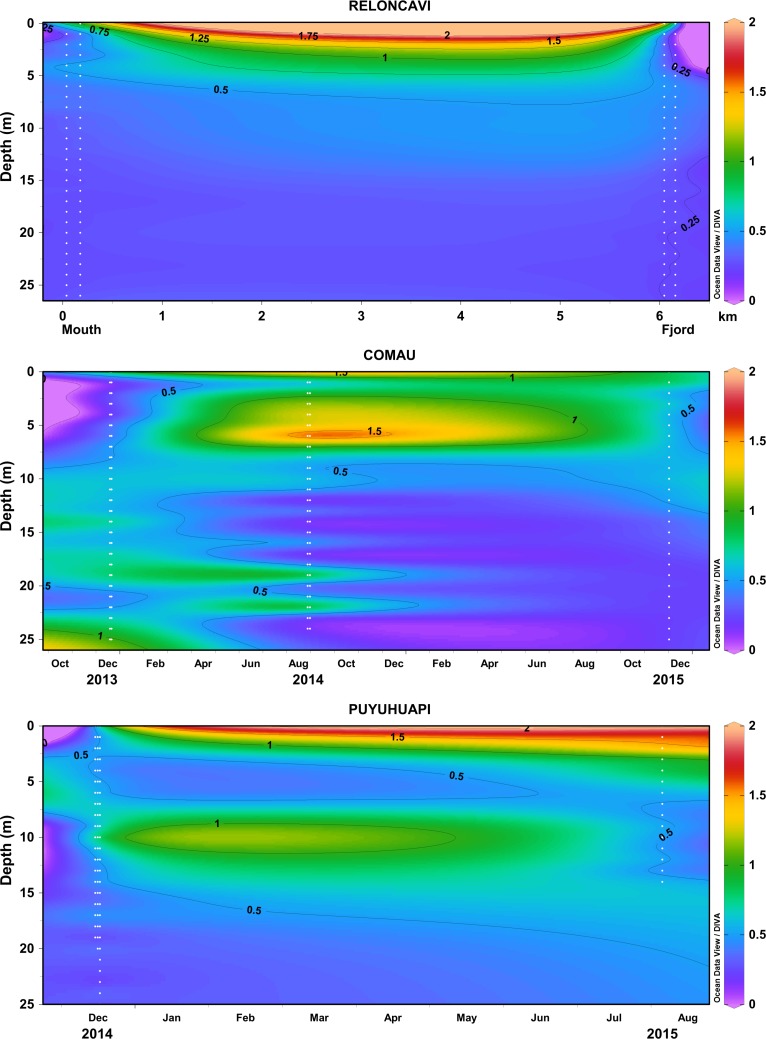
Vertical variation of K_d395nm_ (presented for each one-meter water layer) in the studied North Patagonian fjords, visualized with Ocean Data View software (Schlitzer R., Ocean Data View, odv.awi.de, 2015). The data of different sites (Reloncaví) of measurements are included in order of their geographical location (see map in **Fig 1**; distances not in scale), including all the individual measurements (white dots indicating the measured values; **S2 Data**). For Comau Fjord and Puyuhuapi Channel seasonal variation is presented.

**Fig 8:**
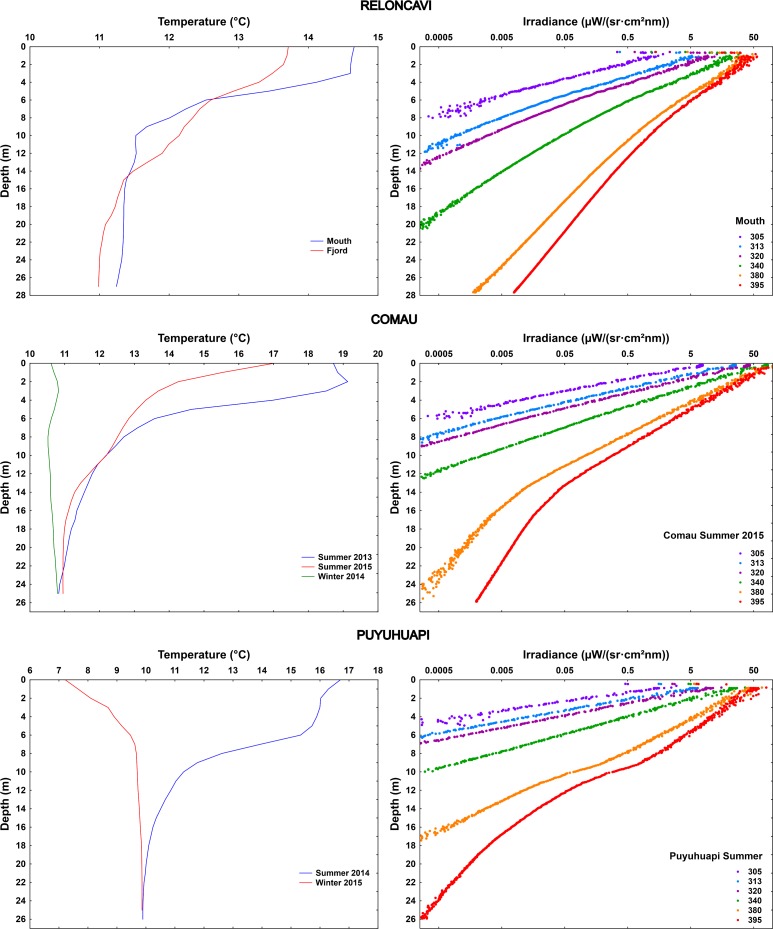
Vertical profiles of temperature (mean of replicate measurements for each location) and underwater solar UV radiation (305–395 nm) (representative example of one site) in the studied fjords in the North Patagonian region.

In Reloncaví Fjord, both study sites within and in the mouth of the fjord showed strong thermal stratification, but only slight optical stratification was observed, mainly at the surface (**Figs 7 and 8**).

**Fig 9:**
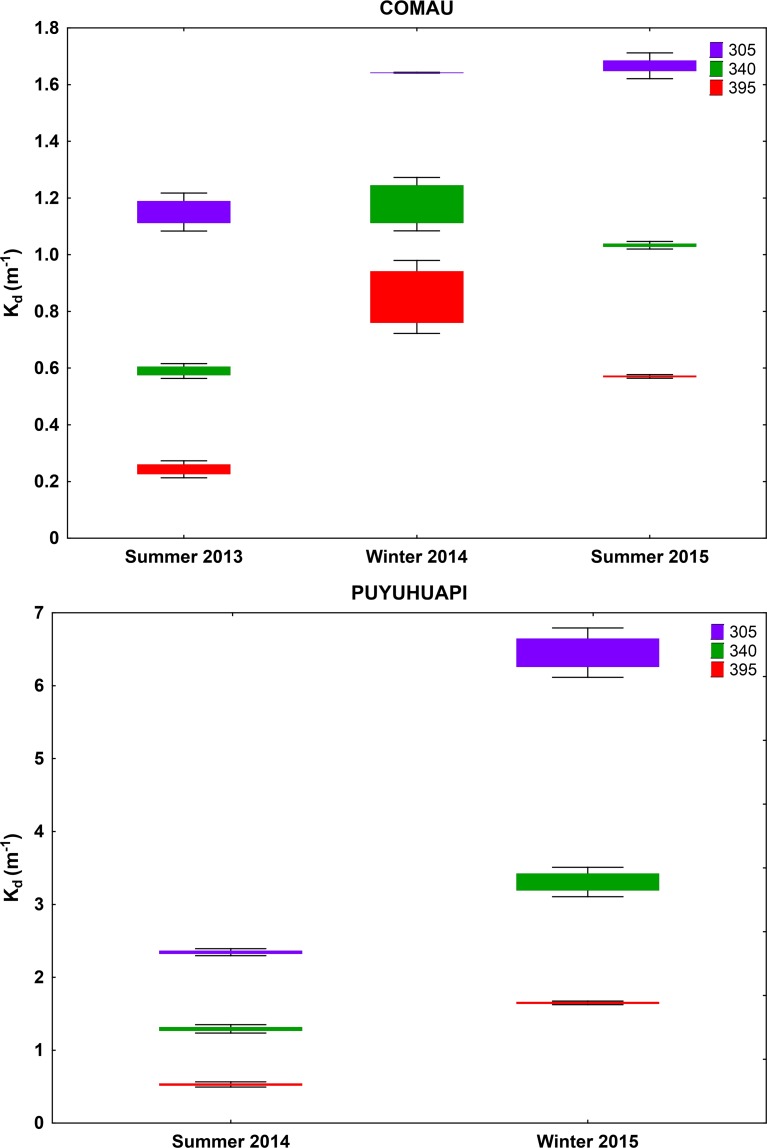
Seasonal variation of coefficient of vertical diffuse attenuation (K_d_) of UV-B (305 nm) and UV-A (340 and 395 nm), derived from the optically homogeneous upper water column in Comau Fjord and Puyuhuapi Channel. Values are means ± S.D., 3 (Comau Summer 2013), 6 (Comau Summer 2015), 2 (Comau Winter), 5 (Puyuhuapi Summer), 3 (Puyuhuapi Winter). Results of multivariate RM ANOVA (Comau) and Hotteling’s T test (Puyuhuapi) for differences in means between seasons are indicated in **S3A and S3B Table**.

In Comau Fjord, temperature in the upper water column reached 17–19°C in summer with cooler (11–12°C) water layer observed below 10 m (**Fig 8**). The Repeated Measures ANOVA indicated that this fjord was characterized by significant seasonal differences in the attenuation of the different wavelengths: lower UV transparency of water was observed in winter than in summer, with significant differences also between summer 2013 and 2015 (**Figs 7 and 9; S3A Table**). Strong optical stratification in the water column was observed in winter (**Fig 7**), although the studied water column was mixed (**Fig 8**). In summer 2013, the water was optically more transparent in the mixed layer, while in summer 2015 higher transparency was observed in the deeper colder zone (**Figs 7–9**). The strong seasonality was also reflected in S_Kd (305-395nm)_ (0.0067–0.0089 nm^-1^ in winter, 0.0116–0.0120 nm^-1^ in summer 2015, 0.0153–0.0188 nm^-1^ in summer 2013) (**Fig 3; S1B Table**).

Also in Puyuhuapi Channel UV attenuation was stronger in winter than in summer, especially the shorter wavelengths (**Figs 7 and 9; S3B Table**). In summer, a layer with higher K_d395nm_ was observed at a depth coinciding with the thermocline (**Figs 7 and 8**). In winter, a layer with higher K_d395nm_ was detected in the upper five-meter (**Fig 7**) coinciding with a cooler water layer. Temperature was around 16°C in the upper six-meter water column in summer, with cooler water (10°C) around 20 m. In winter, cooler upper six-meter water layer (7–10°C) and deeper layer with constant temperature (10°C) prevailed (**Fig 8**). Seasonality was not reflected in S_Kd (305-395nm)_ (0.00142–0.0146 nm^-1^ in winter, 0.0152–0.0166 nm^-1^ in summer) (**Fig 3; S1B Table**).

### Antarctic

The Antarctic study site Fildes Bay showed the highest UV penetration of all the study locations (z_1%313nm_ 10.2–13.3 m, z_1%395nm_ 20.6–34.8 m) (**Fig 2; S1A Table**). S_Kd (305-395nm)_ in Fildes Bay (0.0076–0.0132 nm^-1^) was in the range of the studied estuaries and Comau (summer 2015) and Reloncaví Fjord (**Fig 3; S1B Table**). In Fildes Bay z_10%340_/z_10%305_ (1.7) followed the same order of magnitude as in the other studied areas. However, the UV-A (340 nm) penetrated somewhat deeper in relation to PAR (z_10%PAR_/z_10%340_ 1.9) than in other locations (**Fig 4**).

**Fig 10:**
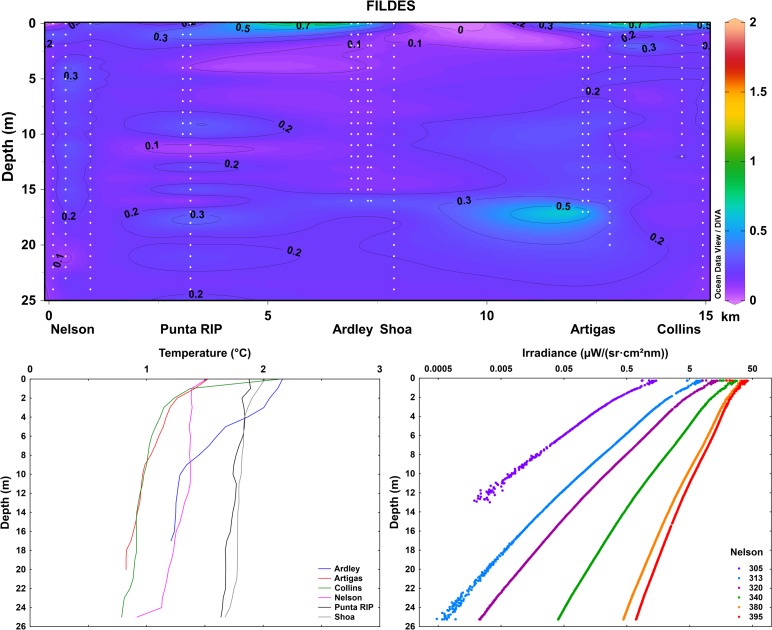
Upper figure: Vertical variation of K_d395nm_ (presented for each one-meter water layer) in the Fildes Bay, Antarctica, visualized with Ocean Data View software (Schlitzer R., Ocean Data View, odv.awi.de, 2015). The data of different sites of measurements are included in order of their geographical location (see map in **Fig 1**; distances not in scale), including all the individual measurements (white dots indicating the measured values; **S2 Data**). Lower figures: Vertical profiles of temperature (mean of replicate measurements for each location) and underwater solar UV radiation (305–395 nm) (representative example of one site) in the studied locations in the Fildes Bay, Antarctica.

Fildes Bay was characterized by relatively high transparency and low temperatures (**Fig 10**). Based on the multivariate repeated measures ANOVA, no differences were detected in K_d_ between sites within Fildes Bay (p>0.05), however, an interaction between sites and wavelengths was evident: shorter wavelengths (305, 313 and 320 nm) measured in sites close to glaciers had K_d_ values significant higher than other sites in the middle of the bay (p<0.05, Tukey LSD; **S2B Table**).

### Multivariate discriminant analysis

**Fig 11:**
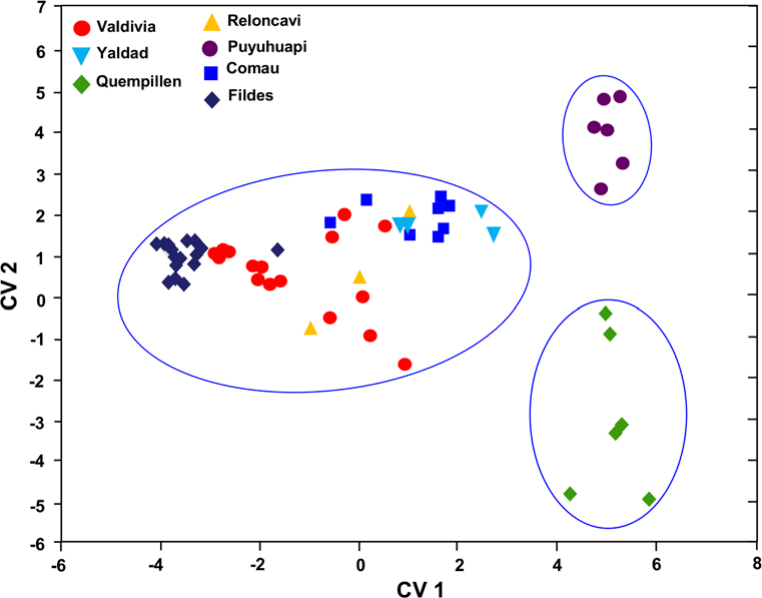
Canonical scatterplot based on discriminant analysis of optical properties of different localities from North Patagonian region and Antarctica. Axes represent first (CV1) and second (CV2) discriminant functions (**S2 Table**).

The discrimination between the seven study locations was significant (Wilks’ lambda = 0.008; *F*_36,204_ = 10.974; p < 0.00001) (**S4A Table**). The standardized coefficients revealed that the variables K_d340_, K_d320_ and K_d305_ contributed most to the overall variability in the CV1 (70.9%), while in the CV2 the overall variability (22%) was marked mostly by K_d320_, K_d340_ and K_dPAR_ (**S4B Table**). The canonical representation revealed that the optical variables in the CV1 and CV2 discriminated well Quempillén Estuary and Puyuhuapi Channel from the other sites (**Fig 11**; **S4C Table**).

## Discussion

### Variation of K_d_ between different water bodies

Multivariate analysis using optical variables discriminated Quempillén Estuary and Puyuhuapi Channel from the other localities. Attenuation of UV radiation (summer) was the strongest in the shallow estuary of Quempillén river and weakest in Fildes Bay, penetration depths (z_1%_) ranging 2–11 m for UV-B (313 nm) and 4–27 m for UV-A (395 nm), respectively. Euphotic zone (z_1%PAR_) in Fildes Bay was 34 m at the maximum. Due to the shallowness (few meters) of the Quempillén Estuary, the euphotic zone reached the bottom. Shallow estuaries like Quempillén experience strong changes in the water characteristic, such as turbidity, resulting from sediment resuspension, as well as influence from catchment area and through tidal regime, creating conditions that are reflected in a strong light attenuation in the water column. Increase in turbidity during low tide can decrease UV penetration 6–7 times and PAR to half in this estuary [5].

Not only the penetration depths, but also the proportions of different wavelengths influence the potential risk. Although in the present study data on water characteristics (e.g. a_d_, CDOM, chlorophyll) were not available, observed differences in S_Kd(305–395 nm)_ (wavelength-dependence of K_d_) suggest site-specific and seasonal differences in the optical properties. Due to that variation of K_d_ for UV and PAR showed distinct patterns in different environments and seasons (depending on the prevailing optical properties), predictions on UV penetration cannot be made based on K_d_ for PAR, information that is more frequently available. This was also emphasized by Barnes et al. [25]. In general, the relationship UV-B/UV-A (305/340 nm) is maintained stable under conditions of normal ozone layer, but under ozone depletion a change in the proportion is generated as UV-B radiation increases [14]. This relationship was somewhat stable in all the study areas. In Mediterranean Sea, the UV-B/UV-A ratio at a 2-m depth was shown to follow the same trend as in the atmosphere, presenting an eight-fold increase in summer [42]. In Fildes Bay, relatively high transparency and deeper UV-B penetration in proportion to longer UV-A/visible wavelengths (UV-B damage vs. photorepair under UV-A/PAR) were observed, suggesting a higher risk of UV damage for organisms in these Antarctic ecosystems as compared to other study sites, together with elevated risk under episodes of ozone depletion.

The comparison of light attenuation between different water bodies is challenging due to logistical difficulties in obtaining comparable data from several locations within a short time frame, as K_d_ is strongly affected by solar zenith angle [43] (influenced by date and time of day). Furthermore, logistical limitations due to climatic and environmental conditions (wind, clouds, waves), e.g. in the Antarctic and in most sites in winter, make it difficult obtain a large number of replicate measurements. Therefore, in the present study data from different dates were used pooled as representatives of summer or winter, however, only data from measurements within a limited range of solar zenith angle (measurements carried out within few hours around solar noon) were accepted in order to minimize its impact on K_d_. Furthermore, the used data are from sunny or partly cloudy conditions without strong waves, which clearly limits the possible days for optimal measurements. Surface waves can generate dispersion and strong fluctuations in the underwater irradiance [41, 44], a phenomenon observed in the upper few meters of water column especially in Puyuhuapi Channel, Reloncaví Fjord and Fildes Bay, affecting the estimation of K_d_ mainly in longer wavelengths (395 nm, PAR), and resulting in changes in K_d_ in the surface.

### Spatial heterogeneity in underwater optics in an estuary and Antarctic bay

As in the comparison of different water bodies, spatial variation in underwater optics (based on calculations of K_d_ within the optically homogeneous upper layer) was also evidenced within study areas. In the estuary of Valdivia river, over two-fold differences in the UV and PAR penetration between measuring sites was observed, strongest attenuation observed in the river mouth area, suggesting a higher impact of e.g. turbidity and organic matter from the river. Optical stratification also reflected differences between the sites.

The results from repeated measures ANOVA performed in Antarctic data indicated lower spatial variation in K_d_ values as compared to Valdivia. Probably, although this area is surrounded by massive glacier fields, these did not affect the local distribution of hydrographic and optical features of the bay. On the other hand, Fildes Bay has low influence of organic matter of plant origin from catchment area (landscape dominated by tundra) and during summer period it is free of ice cover [39], with less impact of runoff from snowfields on the water column. In contrast increased organic matter through ice melting has been related with decreased water transparency in an Arctic fjord [45]. Glacial meltwater impact was reported to result in a 10-fold increase in UV-B, UV-A and PAR attenuation in a subarctic marine system in Alaska [46]. Also floating ice and icebergs can affect mechanically (obstruction or shading) the light penetration [47]. A recent study showed that the biomass of macroalgal-dominated subtidal communities in Fildes Bay increased with distance from glaciers in a gradient of less environmental stress, however, also suggested that environmental factors, including solar radiation, are less important than biodiversity in shaping mesoscale biomass patterns [12]. In more open ocean systems, e.g. along the Gerlache and Bransfield Straits, strong spatial variation in the penetration of UV-B (K_d305nm_ 0.01–0.56 m^-1^) and PAR (0.08–0.5 m^-1^) has previously been reported for Antarctic waters [48].

### Seasonal and vertical heterogeneity of K_d_ in the fjords

Seasonality in underwater optics characterized both Comau Fjord and Puyuhuapi Channel. In both sites UV transparency was higher in summer during a period of stronger thermal stratification, whose patterns coincide with those previously reported for these sites [38, 49–50]. Due to the influence of freshwater, year-round prevailing two-layer water column with low-salinity surface layer is characteristic for both water bodies. In Comau Fjord, a low-salinity upper layer (8–24 PSU) has been described for the upper 2–3 m [50] and halocline for the upper 10 m (18–29 PSU; [49]). Salinity is higher (30–32 PSU) below 15 m [49]. A well-developed pycnocline prevails between 2 and 10 m in spring-summer months [49–50]. In Puyuhuapi Channel, the upper 6-m low-salinity layer is warmer in summer and cooler in winter than the deep water [38]. Salinity in the surface exhibits variation along the channel, and is at its highest in winter (<25 PSU), while in summer salinity is markedly lower when Cisnes river discharges low [38].

Presenting K_d_ individually for each one-meter water layer revealed strong optical stratification i.e. vertical profile of optically non-homogenous layers. This has also been reported for other fjords [51]. In Comau Fjord, contrasting profiles were observed between two summers: in December 2013, more transparent surface water coincided with the warmer low-salinity layer, while in December 2015 (when thermal stratification was slightly weaker) the upper 12-m water column was optically less transparent than deeper water. In Puyuhuapi Channel, less transparent water layer was observed around 6–12 m depth in December, coinciding with the thermocline. In winter, Comau Fjord presented strong optical stratification, despite the lack of thermal stratification. In Puyuhuapi Channel, the cooler low-salinity upper water layer (5–6 m) in winter showed lower transparency. Reloncaví Fjord showed less optical stratification of the studied fjords. A thin, low-salinity surface layer (<5 m near the mouth) has been described also for this fjord [52–53]. Closeness to the mouth of the fjord and Reloncaví Sound likely generates stronger influence of tidal regime in the study sites [9], in comparison to the other studied fjords.

**Fig 12:**
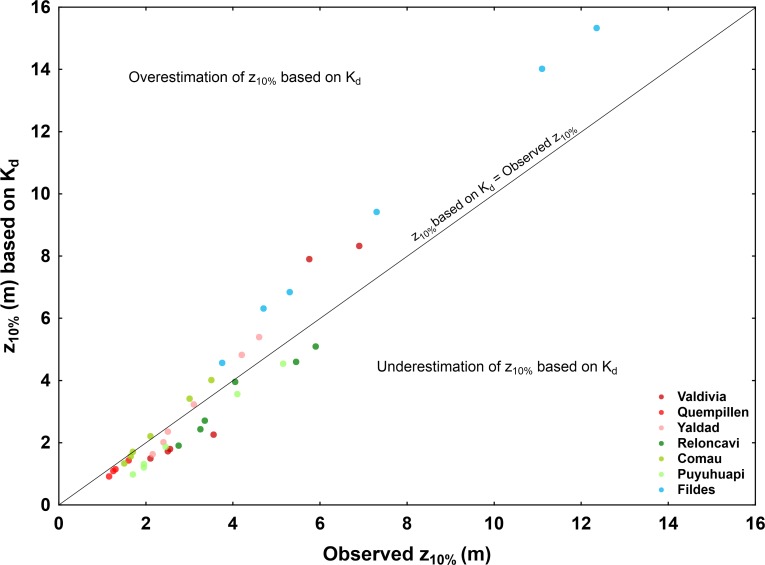
The relationship between the 10% penetration depth (z_10%_) derived from K_d_ (based on log-linear upper water column; S1 Data) and observed from irradiance profiles (depth where 10% irradiance levels were measured; S3 Data). Values of z_10%_ for wavelengths 305, 313, 320, 340, 380 and 395 nm (except for Quempillén where z_10%_ for 380 and 395 nm exceeded total depth) are presented for selected irradiance profiles representing each of the study areas in the North Patagonian estuaries and fjords and Antarctic bay, corresponding to those whose irradiance profiles are given in **Figs 6, 8 and 10** (San Carlos for Valdivia; River site for Quempillén; Mid bay for Yaldad; Mouth for Reloncaví; Summer data for Comau (2015) and Puyuhuapi; Nelson for Fildes Bay).

Overall, the observed patterns (seasonal and vertical) of optical stratification could not be explained by temperature (measured) and salinity (previously described) profiles. Taking into account that inherent optical properties are heterogeneous within water column, optically non-homogeneous layers with distinct patterns are present. For example, in Comau Fjord the vertical variation of nutrients and the composition and amount of phytoplankton and zooplankton, affected by the strong pycnocline [35, 49], are likely reflected in the optical stratification. In the evaluation of UV effects and received UV dose by aquatic organisms it is important to consider the vertical mixing [54]. Overall, the observed optical stratification draws attention to the potential sources of error in the estimations of K_d_ using a wider depth range, which assumes optically homogeneous water column and can overestimate or underestimate the evaluation of penetration depths. In the present study, potential overestimation of penetration depths based on K_d_ could be associated with relatively transparent and optically homogeneous water bodies, such as Fildes Bay in the Antarctic, and in general it increased towards longer wavelengths (380 and 395 nm) (**Fig 12**). In some cases the use of K_d_ led to a slight underestimation of penetration depth, especially at shorter wavelengths (e.g. Puyuhuapi, Reloncaví, Valdivia,). Interestingly, in optically heterogeneous water columns relatively good log-linear fit of irradiance profiles may still be obtained and K_d_ can provide a fairly close estimation of the overall penetration depth (e.g. Yaldad), however, not reflecting the optical variation in different parts of the water column (**Fig 12**).

The marked spatial and temporal heterogeneity of optical water characteristics creates challenges for larger-scale estimations of e.g. primary production [24]. It is evident that field-based estimations of K_d_ cannot provide coverage neither on wide spatial nor temporal scales, as has been recognized previously by other authors [25]. New advances in remote sensing techniques using ocean color from satellites are opening possibilities for evaluations of light attenuation and CDOM absorption in a larger spatial and temporal scale [25, 55–56].

### Potential changes in underwater optics related with changing environmental conditions

Global climate change implies potential contrasting underwater light fields, e.g. lower light availability through increased turbidity from glacier melting [45–46, 57], higher UV transparency via degradation of organic material by UV radiation [58–59] and acidification [60–61]. These changes will challenge the adaptive strategies of photosynthetic organisms, especially the trade-off between UV stress tolerance and light requirements for photosynthesis. For example, in Potter Cove (King George Island), two contrasting phenomena have been reported: while glacier retreat has expanded the areas for settlement of benthic organisms, especially macroalgae [62], enhanced turbidity has strongly modified the vertical setting for photosynthesis, with consequences for the lower limit of distribution of macroalgae [57]. Apparently, these organisms show remarkable abilities to exploit different light climates as has been recently reported in large endemic Antarctic brown algae. Due to their extreme shade adaptation these algae can thrive at depth close to 30–40 m, however, can also be found growing at depth close to 2–5 m, where they exhibit efficient UV stress tolerance [11].

As a consequence of climate warming, the most extreme IPCC near future predictions for the North Patagonian region include increased melting of glaciers resulting in enhanced freshwater runoff as well as increasing rainfall in winter and drought in summer [26]. These scenarios, together with enhanced nutrient loading from intense aquaculture industry in this region [27] with potential impact e.g. on algal blooms and primary production [35], will most likely create changes in bio-optical conditions. Therefore, the optical patterns described in the present survey represent a necessary baseline with improved information for more accurate future predictions. Clearly, simultaneous impact (synergistic or antagonistic) of different factors related to global change, such as enhanced temperature and UV radiation, acidification and freshening, will affect the water transparency in a site-specific manner, but with obvious consequences for pelagic and benthic primary production and thus the entire trophic network. Heterogeneous effects from global change (glacial retreat and enhanced UV radiation) have been proposed also for sub-Arctic marine systems [46]. Organisms, especially in estuarine environments, possess stress tolerance mechanisms in order to cope with the variability and heterogeneity of their physical environment [5]. However, whether the potential for acclimation and adaptation of these organisms to natural perturbations will be enough to respond to larger-scale events driven by global change is among the questions that require attention in order to finally outline predictions for the future of these ecosystems.

## Conclusions

1. UV attenuation was strongest in the estuary of Quempillén river and weakest in Fildes Bay, z_1%_ ranging 2–11 m for UV-B_313nm_ and 4–27 m for UV-A_395nm_. Deeper UV-B penetration in proportion to UV-A/PAR (damage vs. photorepair) suggests a higher risk of UV damage in Antarctic ecosystems, together with elevated risk under episodes of ozone depletion. Differences in wavelength-dependence of K_d_ point to strong site-specificity of the optical properties.
2. Water transparency showed marked spatial variation, which in the estuary of Valdivia river was related to stronger UV attenuation in areas with higher impact of river discharge, while in Fildes Bay higher transparency was a characteristic in almost all the points measured across the bay. Apparently low runoff in the snow-free summer could partly explain this pattern.
3. Strong vertical heterogeneity was characteristic, especially to some of the fjords. Optical stratification did not always follow thermal stratification, but likely reflects the heterogeneity of inherent optical properties. The presence of optically non-homogeneous layers draws attention to the potential sources of error (under- or overestimation) in K_d_ estimations using a wider depth range.
4. Marked seasonality in underwater optics characterized both Comau Fjord and Puyuhuapi Channel. As compared to winter, UV transparency was higher in summer, coinciding with the period of stronger thermal stratification. Significant differences in water transparency were also observed between different summers.

## Supporting Information

S1 Data Dataset on diffuse attenuation coefficient (K_d_), based on the upper log-linear part of the profile, for UV-B (305 and 313 nm), UV-A (320, 240, 280 and 395 nm) and PAR (400–700 nm), 1 and 10% penetration depths (z_1%_, z_10%_) for each wavelength derived from K_d_ values, as well as the slope of the curve ln K_d_ vs. wavelength (S_Kd(305–395 nm)_) for all the study locations in Chilean North Patagonian region and Antarctica. (XLSX)

S2 Data Dataset on diffuse attenuation coefficient (K_d_) of UV-A (395 nm) for each one-meter water column (optical stratification) for all the study locations in Chilean North Patagonian region and Antarctica. (XLSX)

S3 Data Dataset on vertical profiles of temperature (all sites) and irradiance in representative sites (corresponding to those whose irradiance profiles are given in Figs 6, 8 and 10: San Carlos for Valdivia; River site for Quempillén; Mid bay for Yaldad; Mouth for Reloncaví; Summer data for Comau (2015) and Puyuhuapi; Nelson for Fildes Bay) in the study areas in the North Patagonian region and Antarctica. (XLSX)

S1 Table Summary of results of non-parametric analyses for the differences in Z_1%_ (A) and S_Kd(305–395 nm)_ (B) between localities along the Chilean North Patagonian region and Antarctica.
(DOCX)

S2 Table Summary of Multivariate Repeated Measures ANOVA for K_d_ values of different wavelengths (within subject factor) measured in different sites in the Valdivia estuary (A) and Fildes Bay (Antarctica) (B).
(DOCX)

S3 Table Summary of Multivariate Repeated Measures ANOVA and Hotteling’s T^2^ test for the variation in K_d_ values for different wavelengths (within subject factor) measured in winter and summer in Comau fjord (A) and Puyuhuapi channel (B).
(DOCX)

S4 Table Summary of multivariate discriminant analysis indicating the contribution of the different optical variables (A), the standard coefficients of the canonical functions (B) and the discrimination between localities (C).
(DOCX)
