# Supplementary material for: Underwater Optics in Sub-Antarctic and Antarctic Coastal Ecosystems
Source: PLoS One. 2016 May 4;11(5):e0154887. doi: 10.1371/journal.pone.0154887 (PMC4856368; doi:10.1371/journal.pone.0154887)
Supplement: S1 Table — Summary of results of non-parametric analyses for the differences in Z1% (A) and SKd(305–395 nm) (B) between localities along the Chilean North Patagonian region and Antarctica. (DOCX) [file pone.0154887.s004.docx]

**S1 Table**

**Summary of results of non-parametric analyses for the differences in Z_1%_ (A) and S_Kd(305-395 nm)_ (B) between localities along the Chilean North Patagonian region and Antarctica.**

**Table A**

Summary of the differences (Kruskal-Wallis ANOVA) in Z_1%_ of UV-B (313 nm) and UV-A (395 nm) between different localities measured in summer. Results of non-parametric Newman-Keuls multiple comparisons are also indicated. Analysis performed for data shown in Fig 2.

**313 nm**

**i) Kruskal-Wallis test**

|  | Mean Rank | |
| --- | --- | --- |
| Fildes | 53.250 |  |
| Comau (S1) | 31.333 |  |
| Valdivia | 34.000 |  |
| Reloncaví | 31.250 |  |
| Yaldad | 20.400 |  |
| Comau (S2) | 18.000 |  |
| Puyuhuapi | 9.000 |  |
| Quempillén | 3.500 |  |

H_(7, 61)_ =52.681; p< 0.001

**ii) Post hoc Newman-Keuls test**

| Localities | Homogeneous groups | | | |
| --- | --- | --- | --- | --- |
|  | 1 | 2 | 3 | **4** |
| Quempillén |  |  | * |  |
| Puyuhuapi |  |  | * |  |
| Comau (S2) | * |  |  |  |
| Yaldad | * |  |  |  |
| Reloncaví |  | * |  |  |
| Comau (S1) | | * |  |  |
| Valdivia |  | * |  |  |
| Fildes |  |  |  | * |

**395 nm**

**i) Kruskal-Wallis test**

| Localities | Mean Ranks |
| --- | --- |
| Fildes | 51.812 |
| Comau (Summer 2013) | 45.666 |
| Valdivia | 29.437 |
| Reloncaví | 27.333 |
| Yaldad | 22.000 |
| Comau (Summer 2015) | 13.333 |
| Puyuhuapi | 20.000 |
| Quempillén | 3.500 |

H_(7; 60)_ =49.386; p < 0.0001

**ii) Post hoc Newman-Keuls test**

| Localities | Homogenous Groups | | |
| --- | --- | --- | --- |
|  | 1 | 2 | 3 |
| Quempillén |  | * |  |
| Comau (Summer 2015) | * |  |  |
| Puyuhuapi | * |  |  |
| Yaldad | * |  |  |
| Reloncaví | * |  |  |
| Valdivia | * |  |  |
| Comau (Summer 2013) |  |  | * |
| Fildes |  |  | * |

**Table B**

Summary of the differences (ANOVA, post hoc Newman-Keuls) in Box-Cox transformed S_Kd(305-395 nm)_ between localities. Analysis performed for data shown in Fig 3.

**i) One-Way ANOVA**

|  | d.f. | MS | F | P |
| --- | --- | --- | --- | --- |
| Intercept | 1 | 2012.96 | 14031.89 | p< 0.0001 |
| Localities | 9 | 1.26 | 8.77 | p< 0.0001 |
| Error | 55 | 0.14 |  |  |

**ii) Post hoc Newman-Keuls test**

| Localities | Homogeneous groups | | | |
| --- | --- | --- | --- | --- |
|  | 1 | 2 | 3 | 4 |
| Comau (W) |  | * |  |  |
| Quempillén | * | * |  |  |
| Valdivia | * | * |  |  |
| Reloncaví | * | * |  |  |
| Fildes | * |  |  |  |
| Comau (S2) | * |  |  | * |
| Yaldad | * |  | * | * |
| Puyuhuapi (W) |  |  | * | * |
| Puyuhuapi (S) |  |  | * |  |
| Comau (S1) |  |  | * |  |
