# Supplementary material for: Underwater Optics in Sub-Antarctic and Antarctic Coastal Ecosystems
Source: PLoS One. 2016 May 4;11(5):e0154887. doi: 10.1371/journal.pone.0154887 (PMC4856368; doi:10.1371/journal.pone.0154887)
Supplement: S2 Table — Summary of Multivariate Repeated Measures ANOVA for Kd values of different wavelengths (within subject factor) measured in different sites in the Valdivia estuary (A) and Fildes Bay (Antarctica) (B). (DOCX) [file pone.0154887.s005.docx]

**S2 Table**

**Summary of Multivariate Repeated Measures ANOVA for K_d_ values of different wavelengths (within subject factor) measured in different sites in the Valdivia estuary (A) and Fildes Bay (Antarctica) (B).**

**Table A**

Summary of Multivariate Repeated Measures ANOVA for the differences in K_d_ of different wavelengths (within subject factor) measured in different sites along the estuarine system of **Valdivia**. Results of Mauchly test for sphericity, multivariate analysis and post-hoc comparisons of means are indicated.

**i) RM ANOVA**

| Factors | d.f | | MS | | F | P | |
| --- | --- | --- | --- | --- | --- | --- | --- |
| Intercept | 1 | 52.66 | | 534.21 | | p<0.0001 |  |
| Site | 4 | 1.37 | | 13.86 | | p<0.001 |  |
| Error | 11 | 0.10 | |  | |  |  |
| Wavelength | 6 | 1.53 | | 256.57 | | p<0.0001 |  |
| Wavelength x Site | 24 | 0.06 | | 10.72 | | p<0.0001 |  |
| Error | 66 | 0.01 | |  | |  |  |

**ii) Mauchly Sphericity**

|  | W value | Chi-Sqr. | d.f | P |
| --- | --- | --- | --- | --- |
| Wavelength | 0.18 x 10^-6^ | 136.21 | 20 | 0.0001 |

**ii) Multivariate tests for repeated measures**

|  | Test | Value | F | Effect | Error | P |
| --- | --- | --- | --- | --- | --- | --- |
| Wavelengths | Wilks | 0.00 | 2014.21 | 6 | 6 | p<0.0001 |
|  | Pillai's | 1.00 | 2014.21 | 6 | 6 | p<0.0001 |
| Wavelengths x Sites | Wilks | 0.00 | 7.83 | 24 | 22 | p<0.0001 |
|  | Pillai's | 2.36 | 2.16 | 24 | 36 | p<0.005 |

**iv) Homogenous mean groups (Tukey LSD) for the interaction wavelength x site**

| Site | Wavelength | Mean | Homogeneous groups | | | | | | | | | | | | |
| --- | --- | --- | --- | --- | --- | --- | --- | --- | --- | --- | --- | --- | --- | --- | --- |
|  |  |  | | 1 | 2 | 3 | 4 | 5 | 6 | 7 | 8 | 9 | 10 | 11 | 12 |
|  |  |  | |  |  |  |  |  |  |  |  |  |  |  |  |
| San Carlos | PAR | 0.178 | | * |  |  |  |  |  |  |  |  |  |  |  |
| Los Molinos | PAR | 0.183 | | * |  |  |  |  |  |  |  |  |  |  |  |
| Playa Grande | PAR | 0.233 | | * | * |  |  |  |  |  |  |  |  |  |  |
| Playa Chica | PAR | 0.267 | | * | * | * |  |  |  |  |  |  |  |  |  |
| Los Molinos | 390 | 0.290 | | * | * | * |  |  |  |  |  |  |  |  |  |
| Los Molinos | 380 | 0.316 | | * | * | * | * | * |  |  |  |  |  |  |  |
| Playa Grande | 390 | 0.340 | | * | * |  | * | * | * |  |  |  |  |  |  |
| Playa Grande | 380 | 0.381 | | * | * |  | * | * | * |  |  |  |  |  |  |
| Playa Chica | 390 | 0.399 | | * | * | * |  | * |  | * |  |  |  |  |  |
| River mouth | PAR | 0.406 | | * |  | * | * |  | * | * |  |  |  |  |  |
| Los Molinos | 340 | 0.416 | |  | * | * | * | * | * | * | * |  |  |  |  |
| San Carlos | 390 | 0.431 | |  | * | * | * | * | * | * |  |  |  |  |  |
| Playa Chica | 380 | 0.445 | | * | * | * |  | * |  | * |  |  |  |  |  |
| San Carlos | 380 | 0.493 | |  | * | * | * | * | * | * |  |  |  |  |  |
| Los Molinos | 320 | 0.531 | |  |  |  | * | * | * | * |  | * |  |  |  |
| Playa Grande | 340 | 0.532 | |  |  | * | * | * | * |  | * |  |  |  |  |
| Los Molinos | 313 | 0.571 | |  |  |  |  |  | * | * |  |  | * |  |  |
| Playa Chica | 340 | 0.613 | |  |  |  | * | * | * | * | * |  |  |  |  |
| Los Molinos | 305 | 0.648 | |  |  |  |  |  |  |  |  |  |  | * | * |
| Playa Grande | 320 | 0.668 | |  |  | * |  |  |  | * | * | * |  |  |  |
| River mouth | 390 | 0.681 | |  |  |  |  | * |  |  |  |  |  |  |  |
| Playa Grande | 313 | 0.719 | |  |  | * |  |  |  | * | * |  | * |  |  |
| Playa Chica | 320 | 0.759 | |  |  |  | * |  | * |  | * | * |  |  |  |
| River mouth | 380 | 0.775 | |  |  |  |  | * |  |  |  | * |  |  |  |
| Playa Chica | 313 | 0.809 | |  |  |  |  |  | * |  | * | * | * |  |  |
| Playa Grande | 305 | 0.828 | |  |  |  |  |  |  | * |  | * | * | * | * |
| San Carlos | 340 | 0.973 | |  |  |  |  |  |  |  | * |  | * |  |  |
| Playa Chica | 305 | 1.035 | |  |  |  |  |  |  |  |  |  | * | * | * |
| River mouth | 340 | 1.084 | |  |  |  |  |  |  |  | * |  |  | * |  |
| San Carlos | 320 | 1.239 | |  |  |  |  |  |  |  |  | * |  |  |  |
| San Carlos | 313 | 1.287 | |  |  |  |  |  |  |  |  |  |  |  |  |
| River mouth | 320 | 1.289 | |  |  |  |  |  |  |  |  |  | * |  |  |
| River mouth | 313 | 1.327 | |  |  |  |  |  |  |  |  |  |  | * |  |
| San Carlos | 305 | 1.487 | |  |  |  |  |  |  |  |  |  |  |  | * |
| River mouth | 305 | 1.512 | |  |  |  |  |  |  |  |  |  |  |  | * |

**Table B**

Summary of Multivariate Repeated Measures ANOVA for the differences in K_d_ of different wavelengths (within subject factor) measured in different sites at **Fildes Bay** (Antarctica). Results of Mauchly test for sphericity, multivariate analysis and post-hoc comparisons of means are indicated.

**i) RM ANOVA**

| Factors | d.f. | MS | F | P | |
| --- | --- | --- | --- | --- | --- |
| Intercept | 1 | 9.075 | 604.949 | p<0.0001 |  |
| Site | 4 | 0.041 | 2.751 | 0.088 |  |
| Error | 10 | 0.015 |  |  |  |
| Wavelength | 6 | 0.227 | 718.140 | p<0.0001 |  |
| Wavelength x Site | 24 | 0.001 | 4.661 | p<0.0001 |  |
| Error | 60 | 0.0003 |  |  | |

**ii) Mauchly’s Sphericity Test**

|  | W value | Chi-Sqr. | d.f. | P |
| --- | --- | --- | --- | --- |
| Wavelength | 0.92 x 10^-8^ | 143.884 | 20 | p<0.0001 |

**iii) Multivariate tests for repeated measure**

|  | Test | Value | F | Effect d.f. | Error d.f. | P |
| --- | --- | --- | --- | --- | --- | --- |
| Wavelength | Wilks | 0.00047 | 1750.821 | 6 | 5 | p<0.0001 |
|  | Pillai's | 0.99954 | 1750.821 | 6 | 5 | p<0.0001 |
| Wavelength x Site | Wilks | 0.00008 | 10.798 | 24 | 19 | p<0.0001 |
|  | Pillai's | 2.952 | 3.756 | 24 | 32 | p<0.001 |

**iv) Homogenous mean groups (Tukey LSD) for the interaction wavelength x site.**

| Site | Wavelength | | Mean | Homogeneous groups | | | | | | | | | | | | | | | | | | | |  |
| --- | --- | --- | --- | --- | --- | --- | --- | --- | --- | --- | --- | --- | --- | --- | --- | --- | --- | --- | --- | --- | --- | --- | --- | --- |
|  | |  |  | 1 | | 2 | | 3 | | 4 | | 5 | | 6 | | 7 | | 8 | | 9 | | 10 | |  |
| Ardley | | PAR | 0.117 | | * | |  | |  | |  | |  | |  | |  | |  | |  | |  | |
| Ardley | | 390 | 0.133 | | * | | * | |  | |  | |  | |  | |  | |  | |  | |  | |
| Ardley | | 380 | 0.147 | | * | | * | |  | |  | |  | |  | |  | |  | |  | |  | |
| Collins | | PAR | 0.162 | |  | | * | | * | | * | |  | |  | |  | |  | |  | |  | |
| Nelson | | PAR | 0.171 | | * | |  | | * | | * | | * | |  | |  | |  | |  | |  | |
| Punta RIP | | PAR | 0.175 | | * | | * | | * | | * | | * | |  | |  | |  | |  | |  | |
| Collins | | 390 | 0.181 | |  | | * | | * | | * | |  | | * | |  | |  | |  | |  | |
| Collins | | 380 | 0.193 | |  | | * | | * | | * | |  | | * | |  | |  | |  | |  | |
| Artigas | | PAR | 0.196 | | * | | * | | * | |  | | * | |  | |  | |  | |  | |  | |
| Nelson | | 390 | 0.204 | | * | |  | | * | | * | | * | | * | |  | |  | |  | |  | |
| Punta RIP | | 390 | 0.205 | | * | | * | | * | | * | | * | |  | |  | |  | |  | |  | |
| Nelson | | 380 | 0.214 | | * | |  | | * | | * | | * | | * | |  | |  | |  | |  | |
| Punta RIP | | 380 | 0.225 | | * | | * | | * | | * | | * | |  | |  | |  | |  | |  | |
| Artigas | | 390 | 0.227 | | * | | * | | * | |  | | * | | * | |  | |  | |  | |  | |
| Ardley | | 340 | 0.236 | |  | |  | |  | | * | | * | | * | |  | |  | |  | |  | |
| Artigas | | 380 | 0.236 | | * | | * | | * | |  | | * | | * | |  | |  | |  | |  | |
| Collins | | 340 | 0.288 | | * | |  | |  | |  | |  | |  | |  | | * | |  | |  | |
| Nelson | | 340 | 0.310 | |  | | * | |  | |  | |  | |  | | * | | * | |  | |  | |
| Ardley | | 320 | 0.317 | |  | |  | | * | |  | |  | |  | | * | |  | |  | |  | |
| Punta RIP | | 340 | 0.325 | |  | |  | |  | |  | |  | |  | | * | | * | |  | |  | |
| Ardley | | 313 | 0.346 | |  | |  | |  | |  | |  | |  | | * | | * | | * | |  | |
| Artigas | | 340 | 0.347 | |  | |  | |  | | * | |  | |  | | * | |  | | * | |  | |
| Collins | | 320 | 0.362 | |  | |  | |  | |  | | * | |  | |  | |  | | * | |  | |
| Collins | | 313 | 0.388 | |  | |  | |  | |  | |  | |  | |  | |  | | * | | * | |
| Punta RIP | | 320 | 0.415 | |  | |  | |  | |  | |  | | * | |  | |  | |  | | * | |
| Ardley | | 305 | 0.417 | |  | |  | |  | |  | |  | |  | |  | |  | |  | | * | |
| Artigas | | 320 | 0.418 | |  | |  | |  | |  | |  | |  | | * | |  | |  | |  | |
| Nelson | | 320 | 0.420 | |  | |  | |  | |  | |  | |  | |  | |  | | * | |  | |
| Artigas | | 313 | 0.439 | |  | |  | |  | |  | |  | |  | |  | | * | |  | | * | |
| Punta RIP | | 313 | 0.450 | |  | |  | |  | |  | |  | |  | |  | |  | |  | | * | |
| Nelson | | 313 | 0.455 | |  | |  | |  | |  | |  | |  | |  | |  | |  | |  | |
| Collins | | 305 | 0.466 | |  | |  | |  | |  | |  | |  | |  | |  | |  | |  | |
| Artigas | | 305 | 0.485 | |  | |  | |  | |  | |  | |  | |  | |  | | * | | * | |
| Punta RIP | | 305 | 0.525 | |  | |  | |  | |  | |  | |  | |  | |  | |  | |  | |
| Nelson | | 305 | 0.602 | |  | |  | |  | |  | |  | |  | |  | |  | |  | | * | |
