# Supplementary material for: Underwater Optics in Sub-Antarctic and Antarctic Coastal Ecosystems
Source: PLoS One. 2016 May 4;11(5):e0154887. doi: 10.1371/journal.pone.0154887 (PMC4856368; doi:10.1371/journal.pone.0154887)
Supplement: S3 Table — Summary of Multivariate Repeated Measures ANOVA and Hotteling’s T2 test for the variation in Kd values for different wavelengths (within subject factor) measured in winter and summer in Comau fjord (A) and Puyuhuapi channel (B). (DOCX) [file pone.0154887.s006.docx]

**S3 Table. Summary of Multivariate Repeated Measures ANOVA and Hotteling’s T^2^ test for the variation in K_d_ values for different wavelengths (within subject factor) measured in winter and summer in Comau fjord (A) and Puyuhuapi channel (B).**

**Table A**

Summary of Multivariate Repeated Measures ANOVA for the variation in K_d_ values for different wavelengths (within subject factor) measured in summer 2013, winter 2014 and summer 2015 in **Comau fjord**. Results of Mauchly test for sphericity, multivariate analysis and post-hoc comparisons of means are indicated.

**i) RM ANOVA**

|  | d.f. | MS | F | P |
| --- | --- | --- | --- | --- |
| Intercept | 1 | 46.637 | 17282.03 | p<0.0001 |
| Season | 2 | 1.306 | 484.00 | p<0.0001 |
| Error | 7 | 0.003 |  |  |
| Wavelength | 6 | 1.362 | 818.78 | p<0.0001 |
| Wavelength x Season | 12 | 0.032 | 19.66 | p<0.0001 |
| Error | 42 | 0.002 |  |  |

**ii) Mauchly’s Sphericity Test**

|  | W | Chi-Sqr. | d.f. | P |
| --- | --- | --- | --- | --- |
| Wavelength | 0.000038 | 48.622 | 20 | p<0.001 |

**iii) Multivariate tests for repeated measure**

|  | Test | Value | F | Effect d.f. | Error d.f. | P |
| --- | --- | --- | --- | --- | --- | --- |
| Wavelength | Wilks | 0.00026 | 1251.427 | 6 | 2 | p<0.001 |
|  | Pillai's | 1 | 1251.427 | 6 | 2 | p<0.001 |
| Wavelength x Season | Wilks | 0.00031 | 18.731 | 12 | 4 | p<0.001 |
|  | Pillai's | 1.943 | 17.086 | 12 | 6 | p<0.05 |

**iv) Homogenous mean groups (Tukey LSD) for the interaction wavelength x season**

| Season | Wavelength | Mean | Homogeneous groups | | | | | | | |
| --- | --- | --- | --- | --- | --- | --- | --- | --- | --- | --- |
|  |  |  | 1 | 2 | 3 | 4 | 5 | 6 | 7 | 9 |
| Summer 2013 | PAR | 0.206 | * |  |  |  |  |  |  |  |
| Summer 2015 | PAR | 0.256 | * |  |  |  |  |  |  |  |
| Summer 2013 | 390 | 0.257 | * |  |  |  |  |  |  |  |
| Summer 2013 | 380 | 0.327 | * |  |  |  |  |  |  |  |
| Summer 2015 | 390 | 0.571 |  | * |  |  |  |  |  |  |
| Summer 2013 | 340 | 0.577 |  | * | * |  |  |  |  |  |
| Summer 2015 | 380 | 0.668 |  | * | * |  |  |  |  |  |
| Winter 2014 | PAR | 0.669 |  |  | * | * |  |  |  |  |
| Summer 2013 | 320 | 0.820 |  |  |  | * | * |  |  |  |
| Winter 2014 | 390 | 0.851 |  |  |  |  | * |  |  |  |
| Summer 2013 | 313 | 0.894 |  |  |  |  | * |  |  |  |
| Winter 2014 | 380 | 0.900 |  |  |  |  | * |  |  |  |
| Summer 2015 | 340 | 1.033 |  |  |  |  |  | * |  |  |
| Summer 2013 | 305 | 1.117 |  |  |  |  |  | * |  |  |
| Winter 2014 | 340 | 1.178 |  |  |  |  |  | * |  |  |
| Summer 2015 | 320 | 1.353 |  |  |  |  |  |  | * |  |
| Summer 2015 | 313 | 1.441 |  |  |  |  |  |  | * |  |
| Winter 2014 | 320 | 1.461 |  |  |  |  |  |  | * |  |
| Winter 2014 | 313 | 1.622 |  |  |  |  |  |  |  | * |
| Winter 2014 | 305 | 1.642 |  |  |  |  |  |  |  | * |
| Summer 2015 | 305 | 1.666 |  |  |  |  |  |  |  | * |

**Table B**

Summary of the Hotteling’s T^2^ test for the differences in K_d_ values for different wavelengths estimated for summer 2014 and winter 2015 in **Puyuhuapi channel**.

| Wavelength | Mean Winter 2015 | Mean Summer 2014 | t-value | df | P | S.D. Winter 2015 | S.D. Summer 2014 | F-ratio Variances | P  Variances |
| --- | --- | --- | --- | --- | --- | --- | --- | --- | --- |
| 305 nm | 6.453 | 2.344 | 33.216 | 7 | 0 | 0.276 | 0.048 | 32.728 | 0.006 |
| 3013 nm | 5.291 | 1.942 | 111.350 | 7 | 0 | 0.026 | 0.055 | 4.556 | 0.243 |
| 320 nm | 4.817 | 1.790 | 34.889 | 7 | 0 | 0.189 | 0.049 | 14.594 | 0.026 |
| 340 nm | 3.308 | 1.292 | 25.903 | 7 | 0 | 0.164 | 0.057 | 8.268 | 0.069 |
| 380 nm | 2.076 | 0.696 | 70.380 | 7 | 0 | 0.018 | 0.035 | 3.662 | 0.315 |
| 390 nm | 1.648 | 0.529 | 55.321 | 7 | 0 | 0.020 | 0.036 | 3.203 | 0.366 |
| PAR nm | 0.201 | 0.241 | -1.170 | 7 | 0.280 | 0.006 | 0.067 | 117.597 | 0.003 |

T²= -229 x10^16^; F_(7,1)_= -47x 10^15^; p< 0.0001
