# Supplementary material for: Underwater Optics in Sub-Antarctic and Antarctic Coastal Ecosystems
Source: PLoS One. 2016 May 4;11(5):e0154887. doi: 10.1371/journal.pone.0154887 (PMC4856368; doi:10.1371/journal.pone.0154887)
Supplement: S4 Table — Summary of multivariate discriminant analysis indicating the contribution of the different optical variables (A), the standard coefficients of the canonical functions (B) and the discrimination between localities (C). (DOCX) [file pone.0154887.s007.docx]

**S4 Table**

**Summary of multivariate discriminant analysis indicating the contribution of the different optical variables (A), the standard coefficients of the canonical functions (B) and the discrimination between localities (C).**

**Table A**

|  | Wilks' | | Partial | | F-remove | | p-level | | Tolerance | | 1-Tolerance | |
| --- | --- | --- | --- | --- | --- | --- | --- | --- | --- | --- | --- | --- |
|  | Lambda | | Lambda | | (6,43) | |  | |  | | (R-Sqr.) | |
| S_Kd(305-395)_ | 0.0126 | 0.7054 | | 3.2026 | | 0.0103 | | 0.3891 | | 0.6109 | |  |
| K_d305_ | 0.0113 | 0.7897 | | 2.0418 | | 0.0790 | | 0.0675 | | 0.9325 | |  |
| K_d320_ | 0.0125 | 0.7123 | | 3.0969 | | 0.0124 | | 0.0123 | | 0.9877 | |  |
| K_d340_ | 0.0148 | 0.6006 | | 5.0985 | | 0.0004 | | 0.0195 | | 0.9805 | |  |
| K_d380_ | 0.0130 | 0.6884 | | 3.4697 | | 0.0065 | | 0.1000 | | 0.9000 | |  |
| K_dPAR_ | 0.0123 | 0.7237 | | 2.9268 | | 0.0167 | | 0.5744 | | 0.4256 | |  |
| Wilks' Lambda: 0.00892 approx; F (36,204)=11.974 p<0.00001 | | | | | | | | | | | | |

Summary of Wilks’ Lambda and partial Lambda for the contribution of the different variables entered into the multivariate analysis. The smaller the Wilks’s Lambda, the greater is the contribution of the variable to the overall discrimination. Analysis performed for data shown in Fig 11.

**Table B**

Standard coefficients of six canonical functions (roots) for six bio-optical variables measured in different water bodies in southern Chile. Absolute values of coefficients indicate the contribution of each measured variable to the discriminant function. Eigenvalues and cumulative proportion define the proportion of each canonical function to the explained variance. The most significant functions (Root 1 and 2) were used for the canonical representation.

|  | Root 1 | Root 2 | Root 3 | Root 4 | Root 5 | Root 6 |
| --- | --- | --- | --- | --- | --- | --- |
| S_Kd(305-395)_ | -0.089 | -0.323 | 1.169 | -0.828 | 0.000 | -0.637 |
| K_d305_ | 1.290 | 0.032 | 1.631 | -0.634 | 1.161 | 2.955 |
| K_d320_ | 1.928 | 3.967 | -2.910 | 4.775 | -1.482 | -5.336 |
| K_d340_ | -2.611 | -3.673 | -1.136 | -4.530 | 1.273 | 2.728 |
| K_d380_ | 0.091 | -0.413 | 2.550 | -0.334 | -1.790 | -0.088 |
| K_dPAR_ | 0.312 | -0.579 | -0.118 | 0.669 | 0.769 | -0.507 |
| Eigenvalue | 10.510 | 3.311 | 0.691 | 0.164 | 0.106 | 0.038 |
| Cumulative proportion | 0.709 | 0.933 | 0.979 | 0.990 | 0.997 | 1.000 |

**Table C**

Means of canonical variables for the different studied localities that determine the nature of the discrimination within each canonical function.

|  | Root 1 | Root 2 | Root 3 | Root 4 | Root 5 | Root 6 |
| --- | --- | --- | --- | --- | --- | --- |
| Valdivia | -1.231 | -0.458 | -0.468 | -0.148 | -0.217 | 0.215 |
| Yaldad | 2.559 | 1.909 | 0.734 | 1.177 | -0.202 | 0.147 |
| Quempillén | 5.107 | -3.879 | -0.164 | 0.130 | 0.172 | -0.082 |
| Reloncaví | 0.027 | -0.371 | 3.030 | -0.594 | 0.105 | 0.144 |
| Puyuhuapi | 5.048 | 3.248 | -0.601 | -0.377 | 0.481 | 0.072 |
| Comau | 1.112 | 0.970 | 0.035 | -0.204 | -0.486 | -0.316 |
| Fildes | -3.462 | 0.005 | -0.052 | 0.136 | 0.276 | -0.112 |
